# Antidepressant Potential of Chlorogenic Acid-Enriched Extract from *Eucommia ulmoides* Oliver Bark with Neuron Protection and Promotion of Serotonin Release through Enhancing Synapsin I Expression

**DOI:** 10.3390/molecules21030260

**Published:** 2016-02-25

**Authors:** Jianming Wu, Haixia Chen, Hua Li, Yong Tang, Le Yang, Shousong Cao, Dalian Qin

**Affiliations:** 1 Department of Pharmacology, School of Pharmacy, Sichuan Medical University, Luzhou 86646-000, Sichuan, China; chx@lzmc.edu.cn (H.C.); lihua@lzmc.edu.cn (H.L.); tangy1989@yeah.net (Y.T.); shousongc@gmail.com (S.C.); 2 Chengdu Analytical Applications Center, Shimadzu (China) Co. Ltd., Chengdu 86610-063, Sichuan, China; sscyl@shimadzu.com.cn

**Keywords:** chlorogenic acid, *Eucommia ulmoides* Oliver, cerebrospinal fluid, UHPLC-ESI-MS/MS, antidepressant, raphe neurons, serotonin, synapsin I

## Abstract

*Eucommia ulmoides* Oliver (*E. ulmoides*) is a traditional Chinese medicine with many beneficial effects, used as a tonic medicine in China and other countries. Chlorogenic acid (CGA) is an important compound in *E. ulmoides* with neuroprotective, cognition improvement and other pharmacological effects. However, it is unknown whether chlorogenic acid-enriched *Eucommia ulmoides* Oliver bark has antidepressant potential through neuron protection, serotonin release promotion and penetration of blood-cerebrospinal fluid barrier. In the present study, we demonstrated that CGA could stimulate axon and dendrite growth and promote serotonin release through enhancing synapsin I expression in the cells of fetal rat raphe neurons *in vitro*. More importantly, CGA-enriched extract of *E. ulmoides* (EUWE) at 200 and 400 mg/kg/day orally administered for 7 days showed antidepressant-like effects in the tail suspension test of KM mice. Furthermore, we also found CGA could be detected in the the cerebrospinal fluid of the rats orally treated with EUWE and reach the level of pharmacological effect for neuroprotection by UHPLC-ESI-MS/MS. The findings indicate CGA is able to cross the blood-cerebrospinal fluid barrier to exhibit its neuron protection and promotion of serotonin release through enhancing synapsin I expression. This is the first report of the effect of CGA on promoting 5-HT release through enhancing synapsin I expression and CGA-enriched EUWE has antidepressant-like effect *in vivo*. EUWE may be developed as the natural drugs for the treatment of depression.

## 1. Introduction

*Eucommia ulmoides* Oliver (*E. ulmoides*) known as Du-zhong (in Chinese) or Tuchong (in Japanese), is a traditional Chinese medicine (TCM) used as a tonic medicine in China, Japan, Korea, and other countries for a long time [1,2]. *E. ulmoides* has been widely used to tonify the liver and kidney, and strengthen tendons and bones according to the theory of TCM [3]. Pharmacological studies have shown that *E. ulmoides* exhibits many beneficial effects, including neuroprotection [4], bone loss prevention [5], learning and memory improvement [6,7], ameliorating insulin resistance [8], antihypertension [9], antibacterial [10], lipid-lowering and anti-obesity [11,12], treatment of osteoarthritis [2] and so on. Meanwhile, phytochemical studies have displayed the component complexity of *E. ulmoides*, from which 112 compounds have been isolated and identified, including 28 lignans, 24 iridoids, 27 phenolics, six steroids, five terpenoids, 13 flavonoids and nine others [1]. Among them, chlorogenic acid (CGA, 3-*O*-caffeoylquinic acid), a flavonoid with neuroprotection [13,14], cognition improvement, and other pharmacological effects [15,16,17], has been frequently used as the quality control marker for *E. ulmoides* and its preparations. Therefore, CGA, as the mainly active compound of *E. ulmoides* may be used in treatment of various diseases of central nervous system (CNS). Usually, as the most important feature for the agents used in treatment of CNS diseases, the penetration ability of crossing the blood-cerebrospinal fluid barrier (BLB) and blood-brain barrier (BBB) is critical for their therapeutic effect in the CNS [18]. However, CGA does not seem to be beneficial for crossing BLB and BBB due to its good aqueous solubility [19]. Previous study has clearly demonstrated that CGA from *E. ulmoides* has CNS pharmacological therapeutic activities, but it is unknown whether it can penetrate the BLB and BBB or not. A study by Park *et al.* showed that CGA isolated from *Artemisia capillaris* Thunb exhibited a potent antidepressant effect in a mouse model (30 mg/kg/day for 14 day oral administration) [20]. The study supports the idea that CGA may be able to cross the BLB and BBB of mice to display its therapeutical effects.

Depression is a state of affective disorder that can cause deficits in learning, memory and cognition and a major burden on society. These depression-related pathophysiological changes could be induced by excessive exposure to glucocorticoids, whose secretion is regulated by negative feedback loop in response to short-span mild stress [21]. However, this feedback regulation is lost when exposed to major or prolonged stress, causing a significant rise of glucocorticoid levels [22]. A main and potent glucocorticoid, corticosterone (Cort) could decrease serotonin (5-HT or 5-hydroxytryptamine) release and lead to neurodegeneration when chronic exposure to stress levels of Cort [23], which provided a basis for understanding the impairment of 5-HT decrease in depressive illness. Furthermore, clinical studies found that the hippocampal volume and the level of 5-HT were decreased in the patients with major depression [24]. Meanwhile, agents with enhancing 5-HT concentration at the synapse could alleviate the symptoms of depression [25]. Actually, 5-HT is an important monoamine neurotransmitter and can be found in neurons, platelets, mast cells, and enterochromaffin cells. Because 5-HT cannot cross the BBB, the brain synthesizes its own 5-HT which accounts for 1%–2% of the whole 5-HT supply of body, which is exclusively expressed in the dorsal and median raphe of the rostral brain, a heterogeneous region located between the periaquaduct and fourth ventricle of the midbrain [26]. However, numerous evidence indicates that the expression level of synapsin I, a presynaptic phosphoprotein that anchors synaptic vesicles containing neurotransmitters to the actin cytoskeleton in the distal pool, is positively associated with the maturation of 5-HT release mechanisms [27] and structural maintenance of presynaptic terminals [28], as well as neuronal differentiation, axonal outgrowth and synaptogenesis [29]. Those revelations indicate that 5-HT release via synapsin I plays the key role in the depression. As mentioned above, CGA from the extract of *E. ulmoides* is involved various CNS pharmacological and therapeutic activities, but the mode of action and associated mechanism(s) are still unclear. In the present study, we investigate whether CGA can protect neurons from Cort-induced injury and promote 5-HT release through enhancing synapsin I expression in the cultured cells of fetal rat raphe neurons *in vitro* and CGA-enriched water extract from *E. ulmoides* (EUWE) can cross BLB of rats and exhibit antidepressant-like effect in mice *in vivo*.

## 2. Results

### 2.1. CGA Promotes the Cell Growth of Fetal Rat Raphe Neurons in Vitro

**Figure 1:**
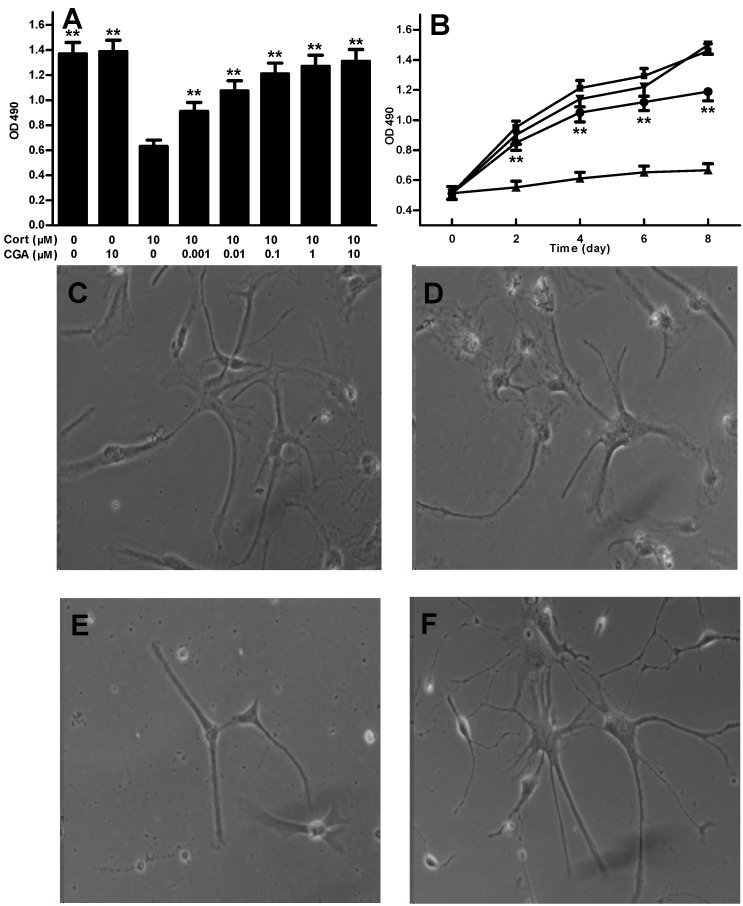
The effects of CGA ± Cort on the cell growth of fetal rat raphe neurons *in vitro*. (**A**) CGA dose response in protection of Cort-induced cell inhibition, CGA 0.001–10 µM and Cort 10 µM; (**B**) Time response curve of CGA in protection of Cort-induced cell inhibition; ■ Control; ▼CGA 1 nM; ▲ Cort 10 µM; and ● CGA 1 nM + Cort 10 µM; (**C**) A representative image of control showing normal neurons; (**D**) A representative image of CGA (1 nM) treatment showing no neurons damage; (**E**) A representative image of Cort (10 µM) treatment showing Cort-induced neurons damage including cell body atrophy, axon and dendrite loss; (**F**) A representative image of co-treatment of CGA (1 nM) and Cort (10 µM) showing prevention of Cort-induced neurons damage by CGA. The control cells were treated with culture medium with 0.1% DMSO. The results are representative of at least three independent experiments run in triplicate and expressed as the mean ± SD. ** *p* < 0.01 *vs.* Cort treatment.

In order to study the protective effect of CGA on Cort-induced cell injury of fetal rat raphe neurons, cell-proliferative assay was performed *in vitro* with cultured cells of raphe neurons treated with 10 µM Cort and 0.001–10.0 µM CGA alone or in combination. As shown in Figure 1, treatment with 10 μM Cort decreased the cell growth of raphe neurons, whereas CGA reversed the Cort-induced decrease of raphe neurons in a dose-dependent (Figure 1A) and time-dependent (Figure 1B) manner. Furthermore, although CGA alone does not affect the cell growth of neurons as similar to the cells of control morphologically (Figure 1C,D), we demonstrate that the inhibition of cell growth by Cort (10 µM) is involved in neuron damage including cell body atrophy, axon and dendrite loss (Figure 1E). Whereas CGA (1 nM) prevents Cort-induced cell damage of raphe neurons by stimulating new axon and dendrite growth (Figure 1F), suggesting the pharmacological effect of CGA on protection of Cort-induced neuron damage is likely to be associated with neurogenesis, which has been proved to be an important factor for antidepressant efficacy [30,31].

### 2.2. Effect of CGA on 5-HT Release in the Cells of Fetal Rat Raphe Neurons in Vitro

**Figure 2:**
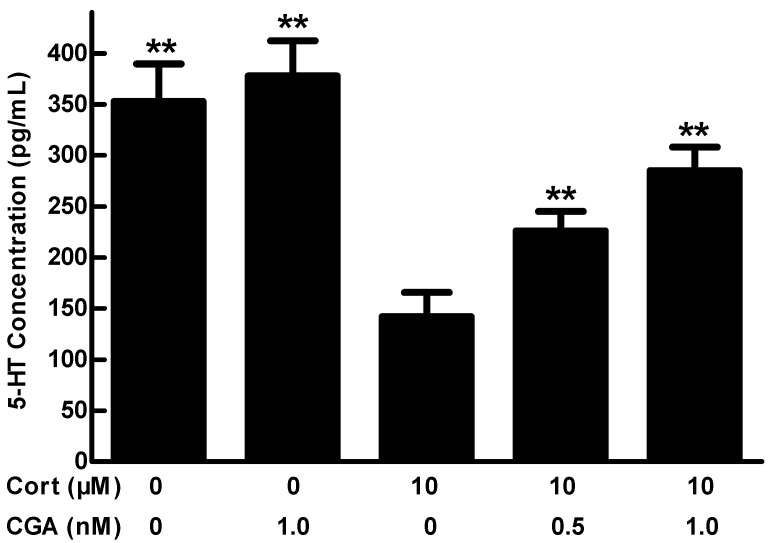
The effects of CGA ± Cort on 5-HT release from the cells of fetal rat raphe neurons *in vitro*. The results are representative of at least three independent experiments run in triplicate and expressed as the mean ± SD. ** *p* < 0.01 *vs.* Cort-treated group. The control cells were treated with culture medium with 0.1% DMSO.

In order to study the effect of CGA on 5-HT release, Enzyme-linked immunosorbent assay (ELISA) was applied to examine the concentrations of 5-HT in the cultured cells of fetal rat raphe neurons after the cells were treated with 10 µM Cort alone or in combination with CGA at 0.5 nM or 1.0 nM. As shown in Figure 2, the level of 5-HT in culture supernatant is significantly reduced after treatment with 10 μM Cort compared to that of the control (*p* < 0.01), however, the reduction induced by Cort is remarkably averted by co-treatment with 0.5 and 1 nM CGA (*p* < 0.01). These results suggest that CGA may promote 5-HT release in the cells of fetal rat raphe neurons.

### 2.3. CGA Enhances the Expression of Synapsin I of the Cells of Fetal Rat Raphe Neurons in Vitro

**Figure 3:**
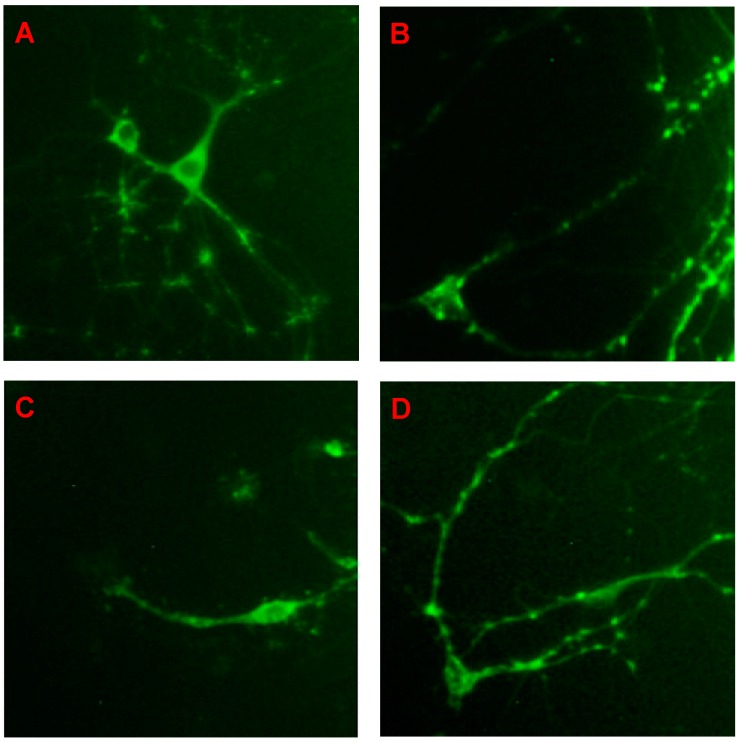
The effects of CGA ± Cort on the expression of synapsin I in the cells of fetal rat raphe neurons. (**A**) Control; (**B**) CGA treatment; (**C**) Cort treatment; and (**D**) Cort + CGA treatment. Notes: Fluorescence microscopy shows the representative images of neurons stained by FITC. The cells were treated with culture medium with 0.1% DMSO (control), or 1 nM CGA ± 10 µM Cort. The results are representative of at least three independent experiments.

**Figure 4:**
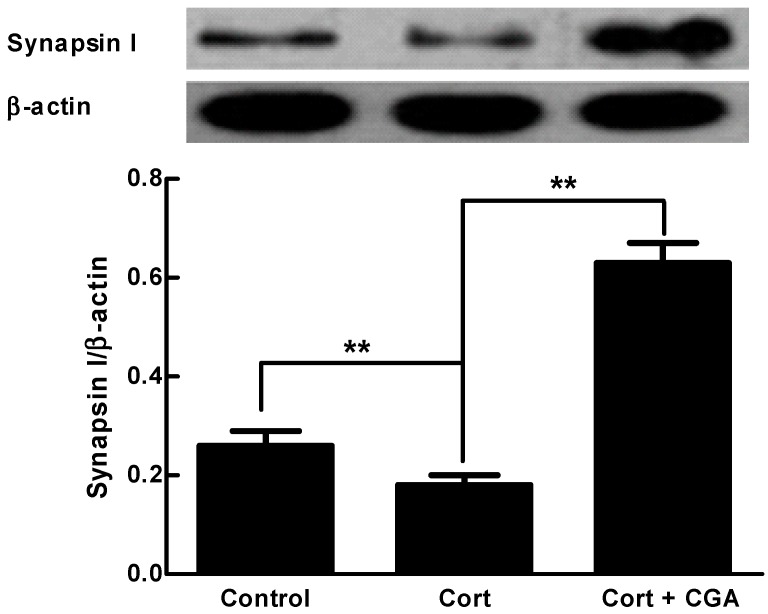
CGA up-regulates protein expression of synapsin I in the cells of fetal rat raphe neurons treated with Cort by western blotting. The cells were treated with culture medium with 0.1% DMSO (control), or 10 µM Cort, or 10 µM Cort + 1 nM CGA. Beta-actin was used as the loading control. The band intensities were quantified by densitometric analysis. The results are representative of at least three independent experiments run in triplicate and expressed as the mean ± SD. ** *p* < 0.01 *vs.* Cort-treated group.

Synapsin I is an abundant synaptic vesicle-related protein that plays a critical role in the regulation of neurotransmitter release [32]. We hypothesized that synapsin I may involve in the neurotransmitter release in neurons. For this purpose, we used anti-synapsin I to examine the location and expression of synapsin I in cultured cells of fetal rat raphe neurons. The fluorescence images shown in Figure 3 illustrate 14-day-old cells of fetal rat raphe neurons, at which point synapsin I distributes to presynaptic terminals in a punctuate manner, concurrent with the formation of synaptic network in normal neurons (Figure 3A). CGA at 1.0 nM has no significant effect on the cells of neurons (Figure 3B). However, the loss of synapsin I puncta and the synaptic network is obvious in the cells of neurons treated with Cort at the concentration of 10 μM (Figure 3C). In contrast to Cort-treated cells of neurons, the loss of synapsin I puncta and the synaptic network are prevented by the co-treatment with CGA at the concentration of 1 nM (Figure 3D). Western blot analysis shows that synapsin I expression levels are significantly down-regulated in Cort-treated cells of neurons compared to that of normal control (*p* < 0.01), while it is significantly up-regulated in Cort plus CGA-treated group compared to that of Cort-treated group (*p* < 0.01), even much higher than that of control, quantified by densitometric analysis (Figure 4). These results indicate that the neurotransmitter release caused by CGA is associated with the expression of synapsin I, suggesting a possible mechanism involved in presynaptic vesicle-related proteins and related signaling pathways.

### 2.4. EUWE Shows Antidepressant-like Effect in the Tail Suspension Test of KM Mice in Vivo

**Figure 5:**
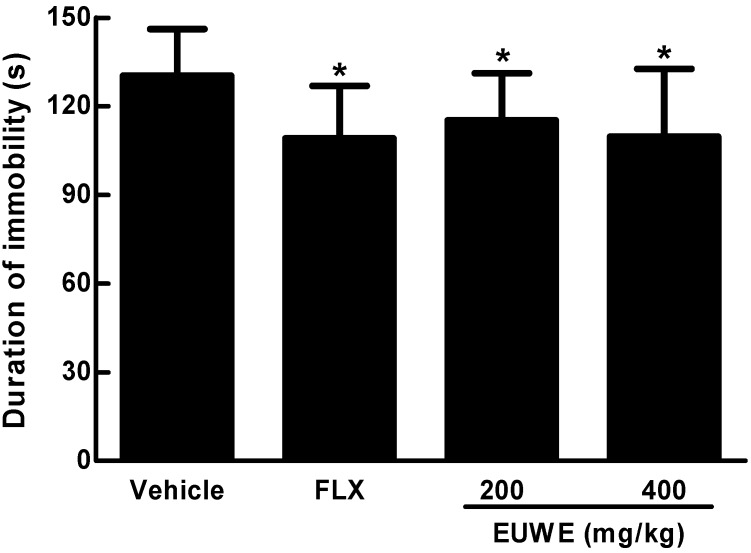
Antidepressant effect of water extract of *E. ulmoides* of (EUWE) in the tail suspension test of mice *in vivo*. The mice were treated orally with EUWE at 200 or 400 mg/kg/day, fluoxetine (FLX) at 8 mg/kg/day and distilled water as vehicle control daily for 7 days. The bars indicate the mean ± SD. The number of mice used for each group was 12. * *p* < 0.05 compared with distilled water (vehicle) group.

The tail suspension test is widely used for screening potential antidepressants. The immobility behavior displays in rodents when subjected to an unavoidable and inescapable stress has been hypothesized to reflect behavioral despair which in turn to reflect similar depressive disorders in human. There is, indeed, a significant correlation between clinical potency and effectiveness of antidepressants [20]. In proof-of- principle study for antidepressant effect of EUWE, we performed the tail suspension test in KM mice with EUWE at 200 and 400 mg/kg/day daily for 7 days, while same volume of distilled water as vehicle control and fluoxetine at 8 mg/kg/day as positive control. As the data shown in Figure 5, the duration of immobility is highest in the distilled water-treated group (control, 130.3 ± 15.9 s), however, it is significantly lessened in fluoxetine (109.1 ± 17.8 s) or EUWE-treated (115.2 ± 16.0 and 109.8 ± 21.9 s, respectively) groups compared to that of control group (*p* < 0.05), indicating EUWE may have antidepressant effects *in vivo*.

### 2.5. Qualitative Analysis of CGA in the CSF of the Rats Treated with CGA-Enriched Water Extract of E. ulmoides

**Figure 6:**
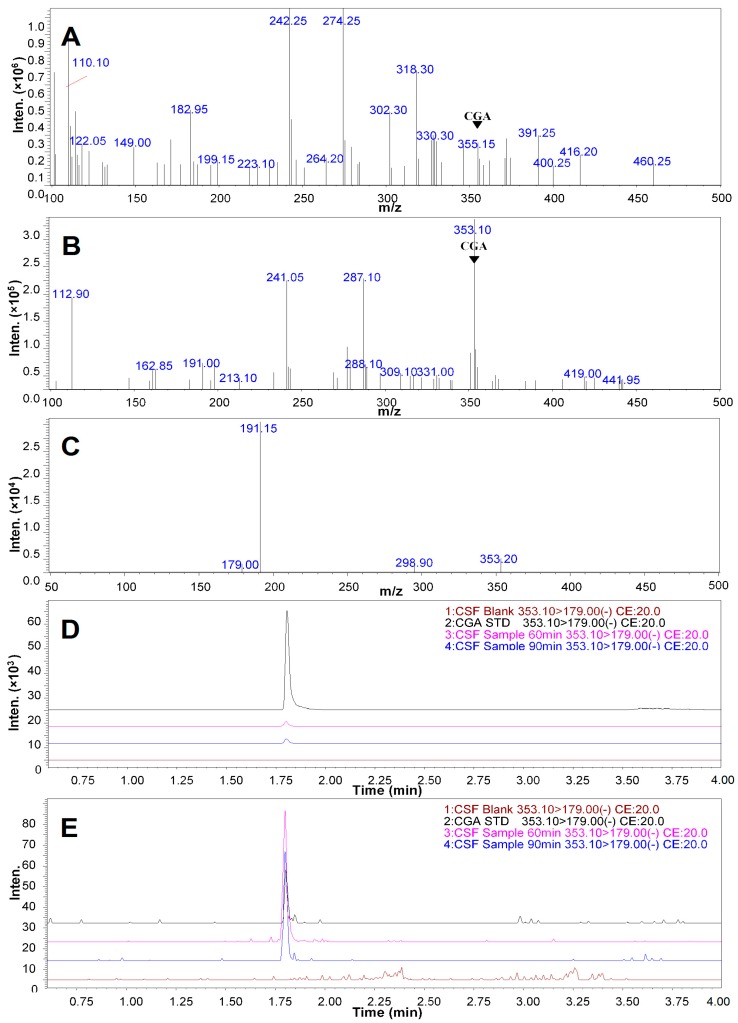
Analysis of CGA and CSF samples in the rats treated with water extract of *E. ulmoides* (EUWE) by UHPLC-ESI-MS/MS chromatogram. (**A**) Positive Scan *m*/*z* 100–500 of CGA; (**B**) Negative Scan *m*/*z* 100–500 of CGA; (**C**) MS/MS spectrum of *m*/*z* 353.10 of CGA in the negative ion mode; (**D**) MRM of channels ESI^−^ 353.10/191.15 for CGA and CSF samples in rat treated with EUWE; (**E**) MRM of channels ESI^−^ 353.10/179.00 for CGA and CSF samples in the rat treated with EUWE. Notes: chromatographic conditions are provided in the Experimental Section. The rats were treated with EUWE at 4.0 g/kg.

**Figure 7:**
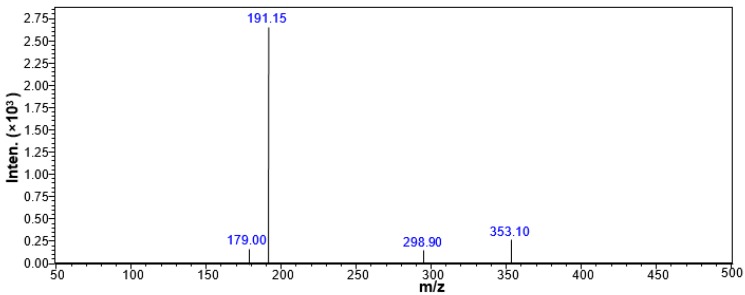
Marker constituents in the CSF of the rats treated with EUWE (4.0 g/kg) identified by ESI-MS/MS spectra in the negative ion mode.

After we demonstrated the pharmacological effects of CGA on the cells of fetal rat raphe neurons, next, we investigate whether CGA can be absorbed into the CSF of the rats treated with CGA-Enriched water extract of *E.* *ulmoides* (EUWE). In order to qualitative analysis of CGA in the CSF of rat, ultra high performance liquid chromatography coupled to tandem mass spectrometry (UHPLC-ESI-MS/MS) was employed in positive and negative scan modes to optimize conditions of mass spectrum (Figure 6A,B). Then MS/MS spectrum of *m*/*z* 353.10 in the negative ion mode was acquired (Figure 6C), and MRM mode was used to monitor both quasimolecular and fragment ions. Therefore, MRM chromatogram of *m*/*z* 353.10 > *m*/*z* 191.15 and *m*/*z* 353.10 > *m*/*z* 179.00 for CGA and CSF samples in the rats treated with EUWE were obtained, respectively (Figure 6D,E). The MRM negative mode was selected due to high sensitivity. As shown in Figure 7, the retention time and mass spectra of the CSF samples in the rats treated with EUWE are similar to that of CGA, indicating the CSF samples may contain CGA.

**Scheme 1:**
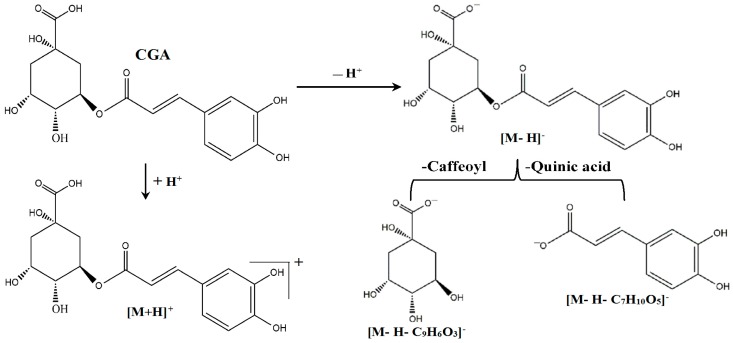
The chemical structures and metabolic pathway of CGA fragmentations identified in the CSF of the rats treated with EUWE (4.0 g/kg).

To further identify whether the CSF of the rats treated with EUWE contains CGA, UHPLC-ESI-MS/MS with both positive and negative ion modes were employed to study the fragmentation behaviors of authentic standard of CGA in ESI-MS/MS at first in order to facilitate the structure characterization of the marker constituent. The marker constituent are identified according to their fragmentation data and comparison with the authentic standard from previous studies [33,34]. The ion fragmentations and structure of marker constituent are shown in Figure 7 and Scheme 1. The quasi-molecular ions [M + H]^+^ and [M − H]^−^ of marker constituent are observed at *m*/*z* 355.15 and *m*/*z* 353.10 in MS spectrum. In the MS^2^ spectrum, the precursor ion [M − H]^−^ at *m*/*z* 353.10 (C_16_H_17_O_9_) fragmented into product ions at *m*/*z* 191.15 (C_7_H_11_O_6_, [M − H − C_9_H_6_O_3_]^−^) and *m*/*z* 179.00 (C_9_H_7_O_4_, [M − H − C_7_H_10_O_5_]^−^). The ion at *m*/*z* 191.15 [C_7_H_11_O_6_]^−^ and *m*/*z* 179.00 [C_9_H_7_O_4_]^−^ are characteristic fragment ions for identifying the structures of caffeoyl and quinic acid from previous reports [35,36,37], which are also observed in the MS spectrum of the authentic standard. Therefore, the data demonstrate that the CSF of the rats treated with EUWE contains CGA and CGA-enriched EUWE can be absorbed into CSF of rats, indicating that CGA can cross the BLB of rats.

### 2.6. Quantitative Measurement of CGA in the CSF of Rat

**Figure 8:**
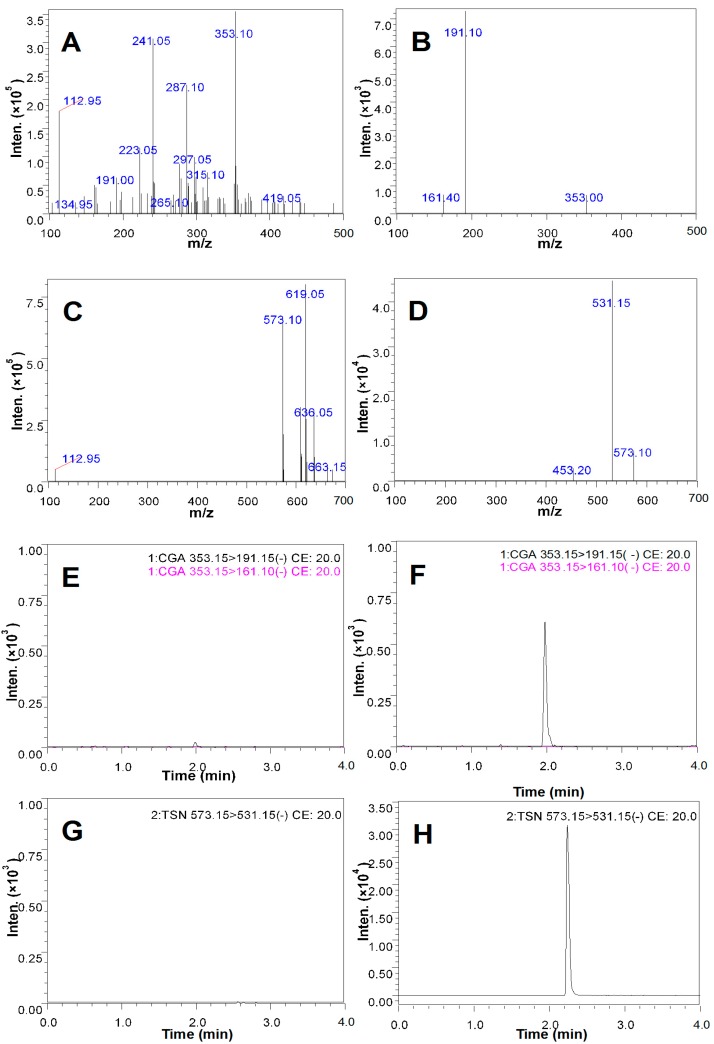
UHPLC-ESI-MS/MS chromatogram of CGA and toosendanin (TSN). (**A**) Negative Scan *m*/*z* 100–500 of CGA; (**B**) MS/MS spectrum of *m*/*z* 353.10 in the negative ion mode; (**C**) Negative Scan *m*/*z* 100–700 of TSN; (**D**) MS/MS spectrum of *m*/*z* 573.10 in the negative ion mode; (**E**) MRM of channels ESI^−^ 353.10/191.15 and 353.10/161.10 for blank CSF sample; (**F**) MRM of channels ESI^−^ 353.10/191.15 and 353.10/161.10 for CSF spiked with CGA; (**G**) MRM of channels ESI^−^ 573.15/531.15 for blank CSF sample; (**H**) MRM of channels ESI^−^ 573.15/531.15 for CSF spiked with TSN. Notes: Chromatographic conditions are provided in the Experimental Section.

In order to achieve a better MS condition, both CGA and toosendanin (TSN, as the internal standard) were examined by negative scan in the MS or MS/MS scan mode (Figure 8A–D). Then MRM mode was used to monitor both quasimolecular and fragment ions, in which channel ESI^−^*m*/*z* 353.10 > *m*/*z* 191.15 was selected for CGA and channel ESI^−^*m*/*z* 573.15 > *m*/*z* 531.15 was selected for TSN. Notably, no endogenous interfering peaks are observed at or near the retention times of CGA and TSN by comparing with blank CSF, and TSN doesn’t contribute to CGA signal (Figure 8E–H), indicating that CGA doesn’t contribute to TSN response and endogenous interfering. Consequently, these results suggest the method has high selectivity for the measurement of CGA.

**Figure 9:**
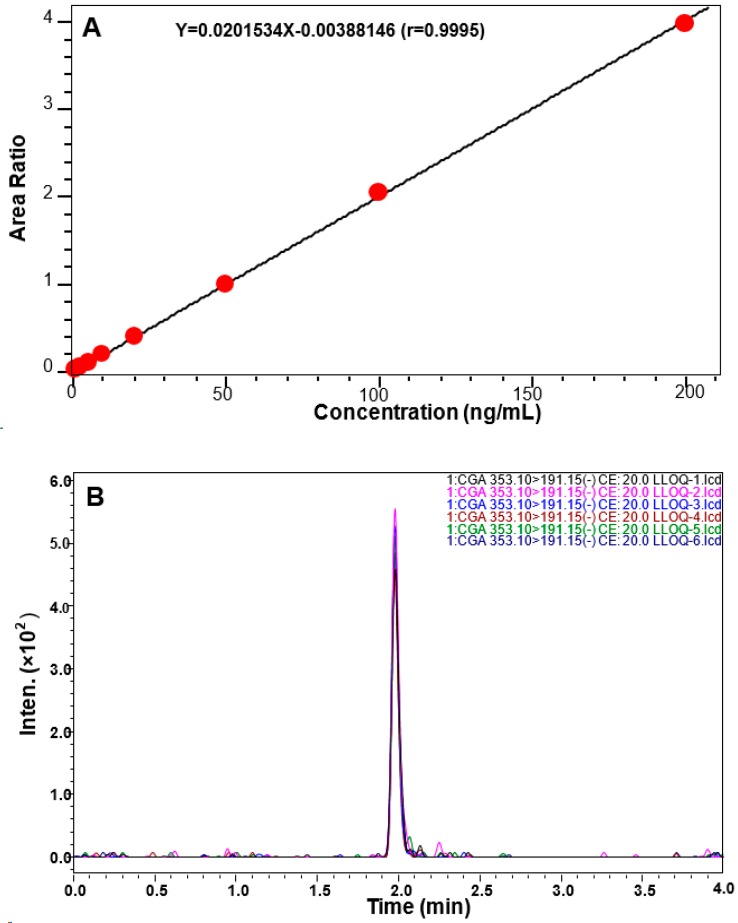
Linear regression calibration curves of CGA (**A**) and the six consecutive MRM of blank CSF spiked with 0.5 ng/mL CGA (**B**) in the CSF of rats.

The calibration curve of CGA in the CSF of rat was constructed by plotting peak area ratios of CGA using the weight (1/C) linear regression. The method shows good linearity over the range from 0.5 to 200 ng/mL with a correlation coefficient r >0.999. The typical calibration curve is presented in Figure 9. The lower limit of quantitation is 0.5 ng/mL. The intra-day accuracy is 111.3% and the relative standard deviation (RSD) of intra-day precision is 7.11% at the concentration of 0.5 ng/mL. In addition, the matrix effect of CGA is 106.15%. Therefore, the method was proved to be sensitive for the measurement of CGA in the CSF of rat.

The established UHPLC-ESI-MS/MS analytical method was subsequently used to determine the CGA concentration in the CSF of the rats treated with EUWE at the dose of 4.0 g/kg and the mean CGA concentrations are 0.41954 ng/mL (1.184 nM) and 0.56224 ng/mL (1.588 nM) for 60 min and 90 min, respectively.

## 3. Discussion

The present study aimed to explore whether CGA can protect from Cort-induced damage and promote 5-HT release through synapsin I expressionin in the cells of fetal rat raphe neurons *in vitro* and cross BLB of the rats orally treated with CGA-enriched EUWE *in vivo*. Our results indicate that CGA can indeed stimulate axon and dendrite growth, promote 5-HT release and enhance synapsin I expression in the cultured cells of fetal rat raphe neurons evidenced by immunofluorescence staining and western blots analysis. More important, our study of *in vivo* antidepressant-like effect in the tail suspension test of mice demonstrated that CGA-enriched EUWE exhibited antidepressant effect (Figure 5). Furthermore, our results also show that CGA could be detected in the CSF of the rats orally treated with CGA-enriched EUWE at 4.0 g/kg and reach to the level of pharmacological effect for neuroprotection, indicating CGA can pass through the BLB of the rats treated with EUWE. Therefore, our findings suggest that CGA and CGA-enriched EUWE may have the potential to become the natural drugs for the treatment of depression. However, their action mode and associated mechanism(s) with anti-depression are still unclear and need to be further investigated.

Synapsin I is a presynaptic phosphoprotein that anchors synaptic vesicles containing neurotransmitters to the actin cytoskeleton in the distal pool [37]. The striking evidence indicates that the expression level of synapsin I is positively associated with the maturation of neurotransmitter release mechanisms [27]. In the present study, we found CGA significantly promoted 5-HT release and stimulated synapsin I expression in the cells of fetal rat raphe neurons *in vitro*, which may provide valuable information for the applications of CGA and CGA-enriched EUWE in potential treatment of depressive disorders in clinic. However, synapsin I controls the fraction of synaptic vesicles available for release and thereby regulates the efficiency of neurotransmitter release by changing its phosphorylation state [38]. The phosphorylation of synapsin I is mediated by multiple protein kinases involved in various signaling pathways, including extracellular signal-regulated kinase (ERK) in mitogen-associated protein kinase (MAPK)/ERK pathway that modulates presynaptic plasticity and learning [39], protein kinase A (PKA) in cAMP-dependent pathway that modulates synaptic vesicle exocytosis [40], and Ca^2+^/calmodulin-dependent protein kinase II (CaMK II) that modulates neurotransmitter release and synaptic plasticity [41]. Therefore, our further investigation into the molecular mechanisms associated with the anti-depression effects of CGA and CGA-enriched EUWE should include the study of phosphorylation of the respective site-specific kinases ERK, PKA and CaMK II in MAPK/ERK, cAMP/PKA, and Ca^2+^/CaMK II pathways, which are upstream of synapsin I.

As we all know, several brain regions including hippocampus have been involved in depression. The hippocampus is an important region of the brain that is in charge of numerous cognitive and behavioral functions and related to the systems of 5-HT and glutamate, which are involved in the mechanism of action of antidepressants so the hippocampus is a key region in which to study depression [42,43]. Moreover, 5-HT has been shown to regulate synaptic neurotransmission in the hippocampus [44]. However, 5-HT is exclusively expressed in the dorsal and median raphe in the brain [26]. Then in order to study the effect of CGA and CGA-enriched EUWE on 5-HT release and its effects in hippocampus simultaneously, the method with a neuronal raphe/hippocampal co-culture *in vitro* should be developed to perform electrophysiological experiments as literature reported [25].

A most important feature for development of antidepressant is the ability to cross BLB and BBB *in vivo* to display its therapeutic efficacy [18]. In the present study, our data demonstrate that CGA-enriched EUWE at 200 and 400 mg/kg/day for 7 days showed antidepressant-like effect in the tail suspension test of mice and CGA from the rats orally treated with CGA-enriched EUWE can be detected in the CSF of rats by UHPLC-ESI-MS/MS analyses, indicating that CGA from EUWE can cross the BLB of the rats. This is the basic for further development of CGA and CGA-enriched EUWE as the antidepressants.

## 4. Experimental Section

### 4.1. Materials

CGA (Lot: 110753-200413) was purchased from National Institutes for Food and Drug Control (Beijing, China). The CGA structure was confirmed on a LCMS-8040 triple quadrupole mass spectrometer (Shimadzu Corporation, Kyoto, Japan). The CSF samples were analyzed by an ultrahigh performance liquid chromatography (UHPLC) system (LC-20 AD, Shimadzu Corporation) coupled to LCMS-8040 triple quadrupole mass spectrometer (Shimadzu Corporation). Acetonitrile (MS grade) and formic acid (MS grade) were purchased from Sigma-Aldrich (St. Louis, MO, USA). Deionized water was prepared using a Milli-Q water purification system (Millipore, Molsheim, France).

Dulbecco’s modified Eagle’s medium (DMEM), neurobasal medium, fetal bovine serum (FBS) and B-27 supplement were purchased from GIBCO Invitrogen (Carlsbad, CA, USA). Polylysine and Cort were purchased from Sigma-Aldrich. Anti-synapsin Ia/b antibody (H-170) was purchased from Santa Cruz Biotechnology (Dallas, TX, USA). Rat 5-HT ELISA kit was purchased from Cusabio Biotech (Newark, DE, USA).

### 4.2. Plant Material and Extraction

The bark of *E. ulmoides* used in this study was purchased from Taiji Group Limited Company (Chongqing, China), and were authenticated by Professor Can Tang at the Sichuan Medical University (Luzhou, Sichuan, China). The dry bark of *E. ulmoides* (100 g) was extracted three times with 1 L distilled water at 100 °C for 60 min each. Then the total extract was concentrated to dryness using a rotary vacuum evaporator and yielded 10.26 g dried extract. This crude EUWE was used for the experiments.

### 4.3. Animals and Sample Collection

Eight-to-ten-week old (body weight 250–300 g), and pregnant (16–20 weeks old and body weight 300–350 g, for primary raphe neuron study) Sprague Dawley rats (SPF Grade, Certificate No. SCXK2013-24) and Six-to-eight-week old (body weight 20–25 g) KM mice (for tail suspension test, SPF Grade, Certificate No. SCXK2013-24) were purchased from Experimental Animal Centre, Sichuan Provincial Academy of Medical Sciences in China (Chengdu, Sichuan, China). All animal experiments were performed in accordance with institutional guidelines and were approved by the Committee on Use and Care of Animals, Sichuan, China (Permit number: SYXK2013-065). All animals were housed under standard environmental conditions and fed with standard diet and water *ad libitum*. The adult rats in the EUWE group were administrated orally with a single dose of 4.0 g/kg EUWE and in the control group with same volume of deionized water before the CSF samples were collected. According to literature [45], rats were anesthetized by 40 mg/kg pentobarbitone, then the atlanto-occipital membrane was exposed by blunt dissection. CSF was collected by lowering a 25-gauge needle attached to polyethylene tubing into the cisterna magna. The pregnant Sprague Dawley rats on embryonic day 15 were used for preparing primary raphe neurons as literature reported [25].

### 4.4. Cell Culture and Treatment

The cells of primary raphe neurons were prepared from pregnant Sprague Dawley rats on embryonic day 15 as previously reported with slight modification [25]. Briefly, cells were gently dissociated with a pasteur pipette after digestion with 0.125% trypsin for 15 min at 37 °C, plated at a final density of 1 × 10^6^ cells/well on polylysine-coated 6-well plates and cultured at 37 °C in a 5% CO_2_ humidified incubator. After 24 h culture, the DMEM medium (with 10% FBS) was replaced by neurobasal medium containing 2% B-27 supplement. For cell proliferative assay, the cells of neurons were seeded into 96-well plates at a density of 3 × 10^4^ cells/well and treated with 10 μM Cort for 24 h, then CGA was applied with different concentrations (0.001, 0.01, 0.1, 1 and 10 μM) or same volume of culture medium containing 0.1% DMSO as control. The assays were performed on the 2nd, 4th, 6th, 8th day using the Dojindo Cell Counting kit-8 according to the instruction supplied by the manufacturer. Absorbance values (490 nm) were recorded in triplicate using M5 Microplate Reader (Molecular Devices, Sunnyvale, CA, USA).

### 4.5. Neurotransmitter Detection

CGA was applied to Cort-pretreated cells of neurons for 5 days. Then the cells were washed 3 times and incubated in KPH buffer (130 mM NaCl, 5 mM KCl, 1.2 mM NaH_2_PO_4_, 1.8 mM CaCl_2_, 10 mM glucose, 1% BSA, 25 mM HEPES, pH 7.4) for 10 min at 37 °C, and subjected to 0.5 nM or 1.0 nM CGA, all treatments were brought up to final concentration in neurobasal medium containing 2% B-27 supplement. After 10 days, the supernatants were concentrated 10-fold using a nitrogen evaporator and the levels of 5-HT were determined using a rat 5-HT ELISA kit. 

### 4.6. Immunofluorescence Staining

CGA (1 nM) was applied to Cort-pretreated cells of neurons for 8 days. The cells were fixed with 4% paraformaldehyde containing 0.05% Triton X-100 for 20 min and rinsed with PBS. After blocked with 4% BSA, the cells were incubated overnight at 4 °C with anti-synapsin I antibody (1:50). Afterward, fluorescein isothiocyanate (FITC) conjugated secondary antibodies (1:100) were applied at room temperature for 1 h. Immunoreactivity was observed with an IX51 fluorescence microscope (Olympus, Tokyo, Japan).

### 4.7. Western Blot Analysis

Neurons were harvested after 7 days CGA (1 nM), and/or Cort (10 μM) or medium with 0.1% DMSO (control) treatment and disrupted in cell RIPA buffer (0.5% NP-40, 50 mM Tris-HCl, 120 mM NaCl, 1 mM EDTA, 0.1mM Na_3_VO_4_, 1 mM NaF, 1 mM PMSF, 1 μg/mL leupeptin, pH 7.5), and then lysates were centrifuged at 12,000 rpm for 15 min at 4°C*.* The protein concentration was determined using the BCA method, after which equal amounts of protein (30 μg) were electrophoresedon 10% density SDS-acrylamide gels. Following electrophoresis, the proteins were transferred from the gel to a nitrocellulose membrane using an electric transfer system. Non-specific binding was blocked with 5% skim milk in TBST buffer (5 mM Tris-HCl, pH 7.6, 136 mM NaCl and 0.1% Tween-20) for 1 h. The blots were incubated with antibodies against synapsin I (1:200) overnight at 4 °C and were washed three times with 1 × TBST. Then, the blots were incubated for 1 h at room temperature with a 1:5000 dilution of horseradish peroxidase-labeled anti-rabbit or anti-mouse IgG and washed three times with 1 × TBST, the membranes were developed by incubation within the ECL western detection reagents.

### 4.8. Tail Suspension Test of Mice

The experiments were performed according to the method of Park *et al.* [20]. Briefly, forty-eight male KM mice were divided into four groups, and the mice were treated orally with distilled water (vehicle control), fluoxetine (FLX) at 8 mg/kg (as positive control), or EUWE at 200 and 400 mg/kg/day with a volume of 0.2 mL/20 g of body weight once a day for 7 days. One hour after the last administration of vehicle, FLX or EUWE, the mice were suspended by the tail to a horizontal ring stand bar (distance from floor 25 cm) using adhesive tape (distance from tip of tail 2 cm). Then the duration of immobility was recorded for the last 4 min during 6-min test session. There are 12 mice for each experimental group.

### 4.9. UHPLC-ESI-MS/MS Analysis

The UHPLC-ESI-MS/MS analyses were performed on a Shimadzu LCMS-8040 UHPLC system comprised of two LC-30AD pumps, a SIL-30AC autosampler with a CTO-30AC column oven, a DGU-20A_5_ degasser, a Shimadzu CBM-20A system controller, a Labsolution LCMS Ver.5.75 workstation, an ESI ion source and a LCMS-8040 mass spectrometer. Chromatographic analyses were achieved at 45 °C with an InertSustain C18 column (GL Science, 2.0 μM particle size, 50 mm × 2.1 mm), using water-formic acid (100:0.05, *v*/*v*) and acetonitrile as the mobile phase A and phase B, respectively. The mobile phase was delivered at a rate of 0.35 mL/min. The injection volume was 10 μL. For the gradient separation, the gradient program was as follows: 5%–5% B at 0–0.8 min, 5%–100% B at 0.8–1.3 min, 100%–100% B at 1.3–2.5 min, 100%–5% B at 2.5–3.0 min, 5%–5% B at 3.0–4.0 min. For mass detection, the mass spectrometer was programmed to carry out a full scan over *m*/*z* 100–500 (MS^1^) and the secondary mass spectrum data were collected by dependence pattern (MS^2^) in positive ion and negative ion detection modes with a spray capillary voltage of 3.0 kV. The detector voltage was 2.04 kV. The desolvation line was heated to 250 °C and the heat block was heated to 450 °C. Nebulizing gas was introduced at 2.5 L/min, and the drying gas was set to 10.0 L/min. Collision-induced dissociation gas pressure was set to 230 kPa. The data analysis was performed using LabSolutions software (version 5.75, Shimadzu).

### 4.10. Statistical Analysis

All data were presented as means ± SD. The statistical significance of the data was analyzed by one-way analysis of variance (ANOVA), and values of *p* < 0.05 were considered statistically significant.

## 5. Conclusions 

In the present study, we demonstrated that CGA plays an important role in neuron protection, promotion of 5-HT release and enhancement of synapsin I expression in the cultured cells of fetal rat raphe neurons. Furthermore, using UHPLC-ESI-MS/MS we also detected and identified CGA in the CSF of the rats after oral administration of CGA-enriched EUWE, indicating CGA could pass through the BLB of rats treated with EUWE *in vivo*. These results may provide important insights into potential discovery and development of CGA and CGA-enriched EUWE as the new antidepressants clinically. However, more studies are needed to further investigate the action mode and associated mechanism(s) of CGA and EUWE as the novel antidepressants. In addition, the *in vivo* antidepressant efficacy of CGA and EUWE should be tested in animal models of depression to validate the results.

## Abbreviations

*E. ulmoides*: *Eucommia ulmoides* Oliver
TCM: Traditional Chinese Medicine
CGA: Chlorogenic acid
BLB: Blood-cerebrospinal fluid barrier
BBB: Blood-brain barrier
Cort: Corticosterone
5-HT: 5-hydroxytryptamine or serotonin
ELISA: Enzyme-linked immunosorbent assay
EUWE: Water extract of *E.* *ulmoides*
FLX: Fluoxetine
TSN: Toosendanin
RSD: Relative standard deviation
ERK: Extracellular signal-regulated kinase
MAPK: Mitogen-associated protein kinase
PKA: Protein kinase A
CaMK: Calmodulin-dependent protein kinase
UHPLC: Ultrahigh performance liquid chromatography
DMEM: Dulbecco’s modified Eagle’s medium
FBS: Fetal bovine serum
